# The Role of Cognition and Social Factors in Competition: How Do People with Intellectual Disabilities Respond to Opponents?

**DOI:** 10.3390/ijerph20032670

**Published:** 2023-02-02

**Authors:** Kandianos Emmanouil Sakalidis, Stein Gerrit Paul Menting, Florentina Johanna Hettinga

**Affiliations:** 1 Department of Sport Exercise and Rehabilitation, Faculty of Health and Life Sciences, Northumbria University, Newcastle upon Tyne NE1 8ST, UK; 2 Department of Human Movement Sciences, University Medical Centre Groningen, University of Groningen, P.O. Box 196, 9700 AD Groningen, The Netherlands

**Keywords:** pacing, time trial, head-to-head trial, sports performance, self-regulation

## Abstract

Exploring pacing behaviour in people with intellectual disabilities (ID) in competition will help to better understand the impact of cognition and social environment in sports, providing support for the shaping of proper inclusive sports environments. The present experimental study aimed to (1) compare the pacing behaviour and performance between people with and without ID who are inexperienced in cycling and (2) investigate how these are influenced by an opponent. Participants with (*n* = 8) and without ID (*n* = 10) performed two randomised 4-km maximal cycling trials, alone and against an opponent. Non-parametric tests for repeated measures data (*p* ≤ 0.05) revealed that people with ID cycled slower, but with higher inter-individual variation (both conditions) and paced themselves differently compared to people without ID when competing against an opponent. In contrast to the previous literature in athletes without ID, the presence of a faster opponent resulted in a decrease in the performance in the participants with ID. The negative influence of the opponent highlights the potential difficulties people with ID experience to adequately use their opponents to enhance their self-regulatory processes and optimize their pacing and performance in maximal exercise trials. Coaches who want to offer inclusive sports environments for people with ID could take these findings into consideration.

## 1. Introduction

Many people with intellectual disabilities (ID) want to progress in their chosen sport, improve their sport-related skills, and become better athletes [1]. Thus, it is the responsibility of an inclusive society to ensure that people with ID can participate and compete in the sport of their choice and master their sports performance [2,3]. However, as many people with ID are prone to being marginalized and stigmatized, their inclusion in sports is still challenging. Thus, inclusive sports environments where people with ID can participate, improve their skills, and compete are limited [4]. Recent findings have revealed that coaches often do not know how to appropriately coach people with ID and how to adapt their behaviour to the specific needs of this population [1,5]. It also seems that coaches are struggling to offer appropriate sports environments to people with ID to develop and improve their sport-related skills [1]. Thus, more research in the sports performance of people with ID could offer the appropriate knowledge to efficiently support and appropriately coach this population [1]. It could also facilitate their inclusion in sports competitions through a better understanding of the impact of cognition on sporting performance [3,6].

People with ID deal with intellectual (IQ ≤ 70) and adaptive skills limitations (practical, social, and conceptual skills), which manifest before the age of 18 years old [7,8]. Their adaptive limitations result in lower sports achievements [9] and impaired technical aspects of sports performance including impaired pacing skills in athletics and cycling [3,6,10].

Pacing in sports is the goal-directed, self-regulatory process in which athletes have to decide how they will distribute their limited energy resources during a race [11,12] and it is characterised as a critical component of sports performance [13,14]. The outcome of this process is termed as the athletes’ pacing behaviour (e.g., cyclists adapt their velocity during the race to reach the finish before their competitor) [15]. During sporting competitions, athletes are constantly looking for information to monitor and self-control their pacing behaviour [16]. The presence of opponents, a well-known competitive scenario in sports, has been shown to impact on this process [17,18]. Through the coupling of perception and action, opponents act as visual stimuli (social affordances) and provide visual guidance and feedback during sports trials and invite athletes to adapt their pacing behaviour [11,17,18,19]. Interestingly, even though we have consistently found that athletes perform better when they compete against an opponent compared to alone, they rated their perceived exertion similarly in both conditions [20,21]. Thus, opponents may act as social placebos, motivate athletes, and help them to better regulate their effort and the negative perceptions of fatigue (through distraction/dissociation) [20,21,22,23,24].

The studies above focused on athletes without ID, while the pacing behaviour of people with ID has received limited attention in the literature. A recent study showed the impact of cognition and social environment in pacing skills [10]. However, it is still unknown as to how the social environment influences time trials at maximal intensity, and if people with ID can adequately respond to their opponents and appropriately pace their actions in a competitive situation. It has been proposed that decision-making in pacing is based on intuitive (responses to the environment) and/or deliberate (pre-planned) responses [25]. Previous research in basketball [26] has revealed that people with ID mainly engage in intuitive decision-making, where the social environment (e.g., opponents) offers visual guidance during the sports activity. Due to this interdependency and the difficulties of most people with ID to regulate their pacing, which has been demonstrated in athletics [6,10], opponents could be a beneficial pacing and performance facilitator for this population. More specifically, they could provide direct pacing feedback during a time trial, act as external distractions, and positively influence the affective responses of people with ID [11,24]. Due to the high levels of ego orientation [27], many people with ID are likely to be motivated by their opponents and use the competition to demonstrate competence [28]. Further investigation of the pacing behaviour of this population in different competitive environments (e.g., with and without opponents) in sports where pacing is critical (e.g., cycling) could help us better understand the impact of cognition in sport performance and how people with ΙD respond to different social situations. It will also provide information to coaches on how to appropriately train and develop the pacing skills and performance of people with ID in an inclusive sport environment [29].

Based on all of the above, this paper aimed to: (1) investigate the performance and pacing behaviour differences of people with and without ID during a maximal cycling time trial and (2) explore the influence of a competitive opponent on the cycling performance and pacing behaviour of people with and without ID. Due to the impact of ID on sports performance [6,30], we hypothesise that people without ID will perform better in all cycling trials compared to people with ID. Due to the cognitive nature of pacing [10,31] and the cognitive deficits of people with ID (e.g., decision-making) [7], we expect that people with and without ID will pace themselves differently when they cycle alone (without an opponent). Moreover, as previous studies have revealed, the presence of an opponent (‘head-to-head’ trial) is expected to positively influence the pacing behaviour and performance of people without ID [17,22,32]. Due to the pacing limitations of people with ID, we hypothesise that the role of the opponent will be even more beneficial in this population, and will offer self-regulatory guidance and assistance to improve the pacing behaviour and performance of people with ID at head-to-head trials [24].

## 2. Materials and Methods

### 2.1. Participants

After obtaining ethical approval by the university’s Ethics Board (reference number: 15,746), participants were recruited through charities, sports clubs, and sports organisations via phone calls and emails. Eight people with (seven males and one female; mean age = 37, *SD* = 8 years; ages ranged from 27 to 52 years) and ten people without ID (six males and four females; mean age = 24, *SD* = 2 years; ages ranged from 20 to 28 years) consented verbally and in writing to partake in our research study. An information letter written in ‘easy read’ language was provided to participants with ID. Due to the vulnerability of the participants with ID, the researchers provided an additional information letter to their legal guardians and also asked for their written consent prior to the participation of their protected person in the study.

Our purpose was to recruit participants with and without ID with similar exercise levels, thus we administered the International Physical Activity Questionnaire-short form (IPAQ-short form) [33]. The IPAQ-short form estimates the participants’ exercise levels and is a valid and reliable instrument [33] for people without ID, while it also seems promising for people with ID [34]. To further ensure that the IPAQ-short form was appropriately completed, participants with ID completed it along with their parents, legal guardians, and/or carers. We recruited only moderate to highly active adult participants who were unfamiliar with (competitive) cycling trials. This means that the participants engaged in some type of physical activity (walking, moderate and/or vigorous intensity physical activity) at least 5 days per week and their weekly sum of MET-mins was more than 600 [33]. It is also worth reporting that the weekly sum of MET-mins was 3195.1 ± 2321.5 for the participants with ID and 2751.6 ± 1131.5 for the participants without ID. All participants engaged in some type of sport and exercise activity such as football, golf, badminton running, and dancing.

Moreover, participants with ID met the criteria for ID diagnosis as set by the British Psychological Society [7]: limitations in adaptive and intellectual functioning (IQ ≤ 70) and limitations in conceptual, practical, and social skills that manifested before the age of 18 years old. The ID diagnosis was established by a certified psychologist from the National Health Service (NHS) in England, and was confirmed by the participants’ parents, legal guardians, and/or carers. Additionally, all the participants with ID were recruited from charities for people with ID. People with severe ID and people who could not understand the activities’ concept and/or instructions were excluded from the study. Prior to testing, the participants (legal guardians and researchers assisted the participants with ID) completed the Physical Active Readiness Questionnaire+ (PARQ+) [35,36,37]. Participants with any related chronic health conditions that may be contraindicated or exacerbated by cycling and/or participants with physical impairments were excluded. Two people with ID were excluded from the study due to their chronic health conditions. None of the 18 participants had a chronic health condition.

### 2.2. Procedure

All participants visited the laboratory on three separate occasions and performed a 4-km maximal cycling trial per visit (three visits in total, with one- or two-weeks difference between each visit). Before each trial, the participants performed a warm-up consisting of seven minutes of sub-maximal cycling. The trials were performed on a Velotron cycle ergometer (Velotron Dynafit, Racermate, Seattle, WA, USA) using a flat and straight course. A projector was used so that the participants could see a real life-sized virtual avatar of themselves on a projection screen. During the first visit, participants were familiarised with the equipment and the protocol and performed a familiarisation trial (without an opponent).

During the second and third visits, participants cycled in two different conditions, alone or against an (virtual) opponent (head-to-head). A computerised randomisation using Excel (Microsoft Office Excel, 2007) was used to determine the order of the condition that each participant cycled in the second and third visits (‘alone’ or ‘head-to-head’). During the ‘alone’ condition, participants were instructed to ‘finish the cycling trial as fast as possible’. During the ‘head-to-head’ condition, participants were instructed to ‘beat the virtual opponent’. To ensure the participants’ perception of a realistic opponent, the virtual opponent’s cycling velocity was based on the participant’s own performance in the familiarisation trial. As a previous study revealed that inexperienced athletes without ID improved their performance by 5% after one visit, the opponents’ cycling velocity was set at +5% of the participants’ cycling velocity at the familiarisation trial, so that the participants were set to compete against a similar level opponent [38]. Before each trial, researchers ensured that the participants were aware of the trial’s goal. Specifically for participants with ID, clear, simplified language was used to explain the trial’s goal. Examples from their daily life along with visual support (pictures) were also used, and the trial started only when the researchers ensured that the participants with ID were fully aware of the trial’s goal. Researchers did not provide any performance feedback (e.g., time or velocity feedback) during the trials.

Researchers recorded the participants’ rate of perceived exertion (RPE) before, after, and twice during the trials (first, second, or third km, based on a computerised randomisation using Excel). A 3-point OMNI scale was used for the participants with ID and a 0–10 OMNI scale was used for the participants without ID [39]. Additionally, the participants’ power output and velocity were recorded (25 Hz) and their average values per 500 m were calculated. The finishing time of the participants was also calculated. Researchers took safety measures to prevent unnecessary illness (COVID-19 related) and injuries and to carefully explain them to the participants. All trials were conducted at an ambient temperature level between 19 °C and 21 °C.

### 2.3. Statistical Analysis

For the analyses, the power output and the velocity were used as the dependent variables. The violation of normality assumption (Shapiro–Wilk test of normality) led us to conduct a series of rank-based non-parametric analyses. To address aim 1 (e.g., if there were performance and pacing behaviour differences between people with and without ID during the trials), we performed non-parametric tests for repeated measures data. More specifically, the ANOVA-Type Statistic (ATS) and the modified ATS were used to test the main and interaction effects [40,41,42]. Analyses were performed separately for each condition. For the analyses, we used the two groups (participants with and without ID) as the between-subjects’ factor and the distance (eight distance points per 500 m) as the within-subjects’ factor.

To address aim 2 (e.g., if the presence of an opponent can influence the cycling performance and pacing behaviour of people with and without ID), we performed a series of ATS. For these analyses, we used the different condition of each group (‘alone’ and ‘head-to-head’) as the between-subjects’ factor and the distance (eight distance points per 500 m) as the within-subjects factor.

To gain further insights into the participants’ performance and pacing behaviour, we used two additional ATS and modified ATS (one for each group) to investigate the velocity differences between the participants at the ‘head-to-head’ condition and their virtual opponents (between-subjects) during the eight distance points of the cycling trials (within-subjects). ATS and modified ATS were also used to explore the RPE differences between the ‘alone’ and ‘head-to-head’ condition (between-subjects) of each group during the three time points (before, during, and after) of the cycling trials (within-subjects).

The between-factor main effects represent the performance differences between groups/conditions. The within-factor main effects represent the pacing behaviour differences within the groups/conditions. If any of the ATS analyses yielded significant interaction effects, pairwise comparisons between groups/conditions (with a Bonferroni-adjusted *p* value) and within each group/condition (with an adjusted *p* value based on Campbell and Skillings modification) were performed to determine where the differences occurred. The statistical analyses were performed on R version 4.1.1, using the ‘nparLD’ and ‘nparcomp’ functions [40,41,42,43]. Using these functions, the relative effects (RTE) were also calculated (increase in the effect indicates an increase in the measured conditions/groups/distances) [40,41,42,43]. A non-parametric version of the coefficient of variation (np-CV) was also used to analyse the inter-individual variation of each group in each condition. The level of significance for all of the analyses was set at *p* ≤ 0.05.

## 3. Results

The finishing time (in seconds) of the participants with LD were *Mdn* = 859.33, *IQR* = 264.38 at the ‘alone’ condition, and *Mdn* = 951.80, *IQR* = 311.91 at the ‘head-to-head’ condition. The finishing time (in seconds) of the participants without LD were *Mdn* = 474.65, *IQR* = 43.46 at the ‘alone’ condition and *Mdn* = 457.87, *IQR* = 38.90 at the ‘head-to-head’ condition. Moreover, one participant with ID (12.5%) and seven participants without ID (70%) won against their opponents during the head-to-head condition.

### 3.1. Pacing and Performance Differences between Groups (ID and Non-ID Group in Each Condition; Aim 1)

In the ‘alone’ condition, the power output, *F*(1, 15.98) = 44.49, *p* < 0.001, and the velocity, *F*(1, 15.89) = 45.90, *p* < 0.001, were significantly lower in participants with ID compared to participants without ID (between-factor main effect). The analysis also showed significant within-factor differences (eight distance points per 500 m) for the power output, *F*(3.25, ∞) = 5.77, *p* < 0.001, and the velocity, *F*(3.37, ∞) = 10.19, *p* < 0.001, but did not reveal significant interaction effects.

**Figure 1:**
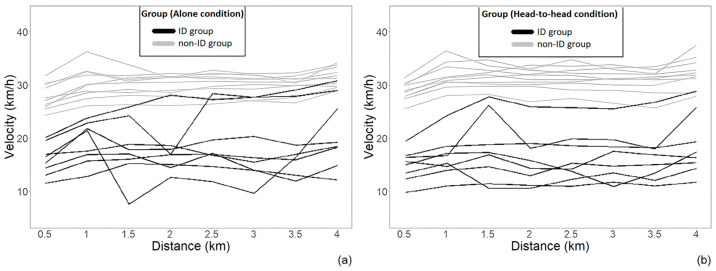
Individual pacing strategies of the ID and non-ID group at the ‘alone’ condition (**a**) and the ‘head-to-head’ condition (**b**).

**Figure 2:**
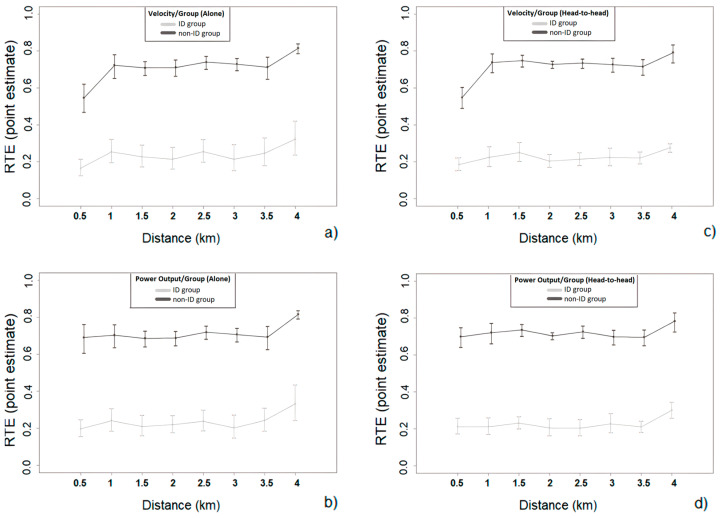
Relative effects and 95% confidence intervals for the velocity and power output of the ID and non-ID group during the ‘alone’ (**a**,**b**) and ‘head-to-head’ condition (**c**,**d**).

In the ‘head-to-head’ condition, the power output, *F*(1, 15.98) = 52.99, *p* < 0.001, and the velocity, *F*(1, 15.73) = 61.27, *p* < 0.001, were significantly lower in participants with ID, compared to participants without ID (between-factor main effect). The analysis also revealed significant within-factor differences (eight distance points, per 500 m) for the power output, *F*(2.51, ∞) = 4.64, *p* < 0.01, and the velocity, *F*(3.10, ∞) = 11.62, *p* < 0.001. Furthermore, it revealed significant interaction effects for the velocity, *F*(3.10, ∞) = 61.27, *p* = 0.01, but not for the power output. Pairwise comparisons revealed significant between-group velocity differences between the participants with and without ID at each time point of the trial (*p* < 0.01). A further analysis showed significant velocity differences only within the ‘head-to-head’ condition of the participants without ID (*p* < 0.001). Multiple comparisons within groups revealed significant within group (participants without ID) differences between 0–0.5-km (*Mdn* = 27.30, *IQR* = 4.10, RankMeans = 27.00) and 1–1.5-km (*Mdn* = 30.61, *IQR* = 2.75; *p* = 0.02, RankMeans = 36.00) and between 0–0.5-km (*Mdn* = 27.30, *IQR* = 4.10, RankMeans = 27.00) and 3.5–4-km (*Mdn* = 31.53, *IQR* = 3.62; *p* = 0.04, Rank Means = 39.37). For the descriptive data and the inter-individual variances of power output and velocity for both groups, in both conditions, refer to Table 1. Please also see Figure 1 for the individual pacing strategies and Figure 2 for the relative effects.

### 3.2. Pacing and Performance Differences between Conditions (‘Alone’ and ‘Head-to-Head’; Aim 2)

The power output and the velocity of the participants with ID were significantly higher in the ‘alone’ compared to the ‘head-to-head’ condition, *F*(1, ∞) = 5.27, *p* = 0.02 and *F*(1, ∞) = 5.58, *p* = 0.01, respectively (between-factor main effect). The analysis did not reveal a significant within-factor main effect nor significant interaction effects for the participants with ID.

**Figure 3:**
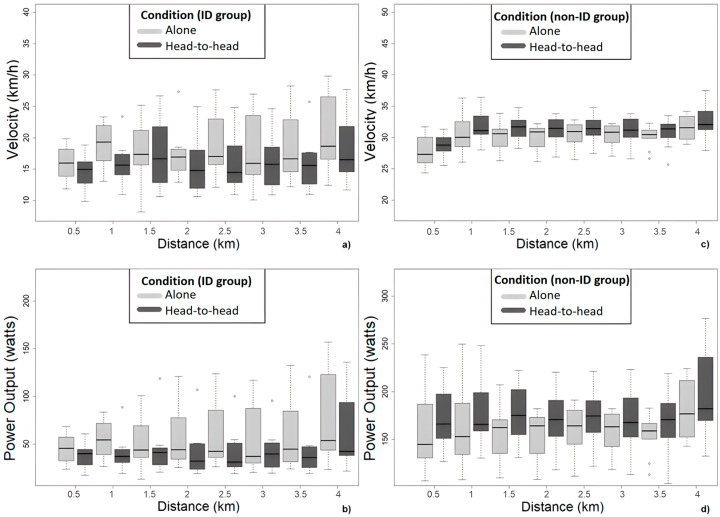
Box plots with the velocity and power output differences per condition (‘alone’ and ‘head-to-head’) for the ID (**a**,**b**) and the non-ID groups (**c**,**d**) at different distance points (° = outliers).

The results showed significant power output, *F*(2.31, ∞) = 5.11, *p* < 0.01, and velocity differences, *F*(2.71, ∞) = 21.90, *p* < 0.001, within the ‘alone’ and ‘head-to-head’ condition of the participants without ID (within-factor main effect). It did not reveal significant between-factor main effects, *F*(1, ∞) = 3.38, *p* = 0.06 and *F*(1, ∞) = 3.01, *p* = 0.08, for the power output and velocity, respectively (which could be considered a trend), nor the interaction effects (see Table 1 for the descriptive data, Figure 3 for the ID and non-ID group box plots respectively, and Figure S1 for the relative effects of both groups).

Moreover, the RPE analysis did not show any significant main effects nor interaction effects for the participants with ID between the ‘alone’ and ‘head-to-head’ condition. On the other hand, for the participants without ID, the RPE level in the ‘alone’ condition (*Mdn* = 5.50, *IQR* = 1.25) was significantly lower, *F*(1, ∞) = 7.94, *p* < 0.01, compared to the ‘head-to-head’ condition (*Mdn* = 5.75, *IQR* = 1.50). The RPE analysis also revealed significant interaction effects, *F*(1.86, ∞) = 4.08, *p* = 0.01 for the participants without ID between the ‘alone’ and ‘head-to-head’ condition. Pairwise comparisons revealed significant RPE differences between the ‘alone’ and ‘head-to-head’ condition only at the final RPE measurement within the trials (*p* < 0.01; second or third km, based on a computerised randomisation). Additionally, a further analysis showed significant RPE differences within the ‘alone’ and within the ‘head-to-head’ condition (*p* < 0.001). Multiple comparisons within groups revealed significant RPE differences (*p* < 0.05) between each distance point of the ‘alone’ and ‘against an opponent’ trial of the participants without ID.

### 3.3. Pacing and Performance Differences between Participants with and without ID and Their Virtual Opponents (‘Head-to-Head’ Condition)

**Figure 4:**
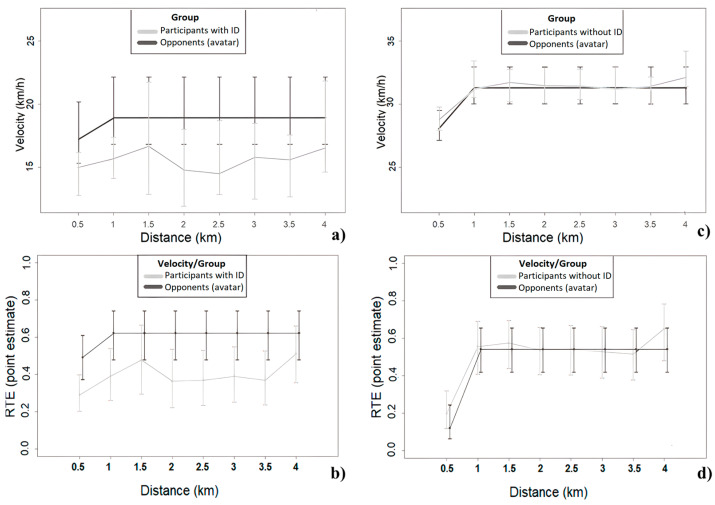
Line graphs with the median velocity differences (+ interquartile ranges) and the relative effects (+ 95% confidence intervals) in the ‘against an opponent’ condition between the participants with (**a**,**b**) and without ID (**c**,**d**) and their virtual opponents (avatars).

In this experimental study, we also compared the pacing and performance differences of the participants (with and without ID) with their virtual opponents (during the head-to-head trial). The comparison between the participants (‘head-to-head’) and their virtual opponents revealed significant velocity differences within the trials for the participants with, *F*(2.70, ∞) = 4.02, *p* < 0.001, and without ID, *F*(2.40, ∞) = 42.16, *p* < 0.001. However, the results did not reveal significant main effects nor interaction effects for both groups (see Figure 4).

## 4. Discussion

The first aim of the study was to explore the performance (between-factor effects) and pacing behaviour (interaction effects) differences of people with and without ID during a maximal cycling trial. The results partially confirmed our first hypothesis (people with and without ID will pace themselves differently within the trials). We found that the power output and the velocity were significantly lower in participants with ID, compared to the participants without ID (in both conditions). Nonetheless, we only found significant velocity differences within the eight distance points (per 500 m) of the ‘head-to-head’ condition of the participants without ID. Moreover, the inter-individual variation was higher in all of the trials of the participants with compared to the participants without ID. The study also aimed to investigate the influence of an opponent on the cycling performance (between-factor effects) and pacing behaviour (interaction effects) of people with and without ID (aim 2). The results did not confirm our hypothesis (the presence of an opponent will positively influence the pacing behaviour and performance of people with and without ID), as they revealed that the presence of an opponent negatively influenced the cycling performance of the participants with ID (slower during the ‘head-to-head’ condition), but not their pacing behaviour. Furthermore, the pacing behaviour of the participants without ID was similar between the ‘alone’ and ‘head-to-head’ condition, while there was a trend toward a faster performance during the latter condition. A possible explanation for not finding significant performance differences as previous studies have demonstrated when people compete against an opponent compared to racing alone [20,21] could be the participants’ experience. The novice participants who were recruited in this study may have responded differently toward their opponents (especially toward a 5% faster opponent) compared to more experienced exercisers/athletes in other studies [15,22].

The difference in pacing behaviour between the participants with and without ID during the ‘head-to-head’ condition and the negative influence of an opponent on performance only for the ID group could further support the suggestion that people with ID engage mainly in intuitive decision-making in pacing and sports [26]. It could also indicate that the majority of people with ID perceive and respond differently to the social environment during a maximal trial compared to people without ID [11]. People with ID experience deficits in interpreting and judging other peoples’ behaviour, social interaction difficulties, increased anxiety, and social phobia [7,44,45,46]. These important social cognition processes could lead people with ID to analyse a social situation, respond to the environmental cues, and regulate their actions differently compared to people without ID [11,24,47].

Opponents could be characterised as self-regulatory, pacing, and performance facilitators [24]. In people without ID, the presence of an opponent is theorised to act as a goal-setter, facilitate the self-monitoring skill, increase motivation, and help improve performance [24]. However, a lot of people with ID may not be able to properly use the opponents as pacing and performance feedback facilitators during a maximal trial and may find the goal (beat a faster opponent) challenging and confusing [3]. As people with ID make slow and inaccurate decisions during a sporting activity [48], the presence of an opponent may be a challenging component that makes their pacing and sports performance development more complicated in competitive sports. These findings indicate that pacing is a cognitive element, and reveal the impact of ID on pacing behaviour, sports performance, and competition.

Motivation could also partially explain why participants with ID performed slower when they competed against a faster opponent. In people without ID, opponents act as goal setters and motivate them to compete and reach their pacing and performance goals [3,27]. People with ID, however, present high controlled motivation [1] and ego orientation [27]. This means that they engage in actions mainly for externally referenced reasons such as to demonstrate competence (e.g., beating an opponent) [28]. Thus, they may abandon the goal to win against an opponent who is 5% faster as they could be perceived as a challenging and unreachable situation. Menting et al. [38] revealed that inexperienced athletes without ID improved their performance by 5% after one visit; however, this may not be realistic for people with ID who experience impaired learning skills [7]. Indeed, during the ‘head-to-head’ trial, most of the participants with ID were always behind their opponents. Until 1.5-km, most of the participants with ID tried to reach their opponents, but after that distance point, their velocity gradually decreased (Figure 3). Even if they increased their velocity during the last km of the trial, it was not enough to reach their opponents’ velocity and overtake them (Figure 4). At a situational level, the person may be susceptible to judging competence, evaluating success and failure, and continue engaging in sporting activities based on social comparisons [49]. The negative social comparisons that many people with ID experience in their daily lives decrease their self-esteem and impact on their mood [50,51]. As the pacing behaviour of people with ID in sports seems to depend on visual affordances (e.g., the opponents) [26], their inability to outperform their opponents in the ‘head-to-head’ condition may negatively influence their motivation, emotional state, and performance [49]. For instance, a similar RPE level but a significant slower velocity at the ‘against an opponent’ compared to the ‘alone’ condition could be an indication of the emotional challenges that many people with ID are dealing with when they compete against a faster opponent and their difficulties in using the opponents as external distractions [24]. The competitive environment could be a challenging situation for people with ID [1], which requires further investigation.

Another explanation of the performance differences of participants with ID between the two conditions could be the lack of experience that people with ID have in performing maximally and competing against an opponent [1]. Despite the aforementioned challenges of the competitive environment, many people with ID want to participate and compete in sports and improve their sport-related skills [1]. The lack of social support and financial resources, however, restricts their access to sporting opportunities [52]. Due to their ID, they may be actively excluded from sports programs and competitions without appropriate adaptations being made or alternative sporting opportunities being provided [52]. Moreover, their coaches may underestimate the athletic identity that people with ID wish to develop [1]. Coaches reported to focus more on sporting participation and less on the performance development of athletes with ID may provide less maximal, competitive sports activities [1]. This could be an indication that coaches of athletes with ID may adopt different standards of sporting success (ableism) and consider the maximal, competitive sports activities as ‘surprising’ and ‘paradoxical’ for this population [1,53]. To properly promote positive sports experiences and achieve a balance between participation-focused and performance-focused approaches, it is important for coaches to listen to the sporting aspirations of people with ID and respond to them accordingly [1].

Some of the pacing and performance differences between the groups (participants with and without ID) were observed in both conditions (‘alone’ and ‘head-to-head’). For instance, participants with ID demonstrated higher inter-individual variations in both trials compared to participants without ID. This might occur due to the inability of many people with ID to maintain a pre-planned steady pace [10] and confirms the previous studies in which they demonstrated high inter-individual variations in exercise-related activities such as technical proficiency [54], physical fitness [55], and reaction time [56]. Additionally, even though we recruited participants with similar physical activity levels who were novices in cycling, participants with ID were cycling significantly slower in both conditions compared to the non-ID group. In addition to the cognitive, self-regulatory, and pacing differences between the groups that were above-mentioned, this could partially explain the performance differences, aspects that are frequently identified in people with ID such as low muscle strength, low voluntary activation levels, and the lack of motivation to use the maximum effort in sports may also contribute [57,58]. Moreover, the age of the participants with ID (older than the participants without ID) might influence their performance and pacing behaviour [15,59].

All of the information above could be useful for coaches who want to include people with ID in sports and improve their performance. With the appropriate adaptations, coaches could promote an inclusive sports environment where athletes with ID will have the opportunity to practice and develop their self-regulatory, pacing, and sports performance skills [5]. For instance, in sub-maximal activities, the introduction of another person could provide a visual stimulus that improves the exercisers’ attention and positively influences the exercise behaviour [24]. In maximal trials, however, the competition against faster opponents could be a stressful situation [1] that negatively influences the perception and the action of people with ID and leads them towards a deteriorated performance. Thus, coaches who engage people with ID in this type of competitive, mastery-avoidance situation may undermine peoples’ sports motivation and negatively influence their further engagement in sports [1]. Coaches who aim to introduce competitive environments to people with ID could provide fewer external cues (e.g., social affordances), engage them in mastery competitive experiences (e.g., compete against a slower opponent), introduce less stressful competitive situations (e.g., competitions against peers), and provide additional supportive strategies tailored to their needs and motivations [1,24]. However, it should be clarified that coaches should acknowledge that each person with ID has a unique personality, and that they should adapt their coaching strategies to each person’s needs [5]. For instance, coaches should support the fulfilment of the psychological needs of people with ID (e.g., provide choices to people with ID, trust their capabilities, and give them the opportunity to express their needs in a supportive environment) [1,5]. This supportive approach could enhance the autonomous motivation of this population and inspire their further engagement in sports [16,24]. It is worth mentioning that the preliminary findings were presented to the participants with ID and their coaches/carers, and their possible meanings were discussed. Participants with ID were encouraged to comment on the findings and give their opinion about the appropriate ways to coach them and interact with them in sports and exercise settings.

The study was subject to limitations. Even if groups were matched based on the participants’ physical activity levels, the participants with ID were older, which might influence their fitness ability and their capacity to appropriately pace their actions during a maximal trial [15,59]. Moreover, competing against a 5% faster opponent may have been quite challenging for the people with ID. Thus, future studies should further explore the influence of different competitive environments in the pacing behaviour and performance of people with ID. For example, competition against a slower opponent could be a less stressful competitive situation for novice athletes with ID that positively influences the athletes’ emotions, pacing, and performance. As adequate pacing can enhance the exercise engagement of people with disabilities and chronic diseases [60,61], a further investigation of the role of the social environment (e.g., peers, significant others) in physical activity of people with ID could be fruitful. More specifically, research should further explore if and how the social environment can help people with ID to better regulate their physical activities, reduce their fatigue perceptions, and ensure a long-term engagement in an active lifestyle. Moreover, waist-worn triaxial accelerometers, proxy, and self-reporting measures could be used to assess the physical activity patterns and levels of people with ID within the day [62]. Then, the relationships of the physical activity patterns/levels with social support and guidance could be explored to further explore the importance of the social environment in the sporting participation and physical activity engagement of individuals with ID.

## 5. Conclusions

This experimental study explored the pacing behaviour and performance of people with and without ID during different competitive conditions (‘alone’ and ‘head-to-head’). It seems that people with ID cycle slower compared to people without ID and they pace their races differently when they compete against an opponent. Moreover, people with ID may not be able to pace themselves appropriately when a faster opponent is present. The significantly faster power output and velocity during the ‘alone’ compared to ‘head-to-head’ condition seem to indicate that the pacing behaviour of people with ID is significantly influenced by visual affordances. This independency though, may negatively influence the self-regulatory behaviour, motivation, and emotions of people with ID, revealing the complexity of competitive environments in non-elite athletes. Coaches who wish to include people with ID in sports environments could take these findings into consideration and explore less cognitive demanding approaches to improve the athletes’ self-regulatory, pacing, and performance skills. This experimental study could promote the participation of people with ID in sports competitions.

## Figures and Tables

**Table 1:** Descriptive statistics of power output (Watts) and velocity (km/h) for the ID and non-ID groups in the ‘alone’ and ‘head-to-head’ condition (4-km).

Variable	Source	Mdn	IQR	Min	Max	np-CV (%)
Power output						
	ID group (alone)	45.05	42.01	28.71	107.93	14.51
	Non-ID group (alone)	161.49	40.46	117.42	197.31	12.93
	ID group (head-to-head)	36.13	23.63	19.76	102.83	21.60
	Non-ID group (head-to-head)	176.77	43.71	122.17	214.26	12.42
Velocity						
	ID group (alone)	16.77	6.29	13.74	25.56	9.54
	Non-ID group (alone)	30.33	2.72	26.70	32.58	4.63
	ID group (head-to-head)	15.13	5.37	11.07	24.58	16.33
	Non-ID group (head-to-head)	31.45	2.66	27.04	33.67	4.28

Note. Mdn = median, IQR = interquartile range, Min = minimum, Max = maximum, np-CV = non-parametric version of the coefficient of variation.

## Data Availability

The data presented in this study are available upon request.

## Supplementary Materials

The following supporting information can be downloaded at: https://www.mdpi.com/article/10.3390/ijerph20032670/s1, Figure S1: Relative effects and 95% confidence intervals for the velocity and power output of the ID (a,b) and non-ID groups (c,d) during the ‘alone’ and ‘head-to-head’ conditions.
