# Supplementary material for: The Role of Cognition and Social Factors in Competition: How Do People with Intellectual Disabilities Respond to Opponents?
Source: Int J Environ Res Public Health. 2023 Feb 2;20(3):2670. doi: 10.3390/ijerph20032670 (PMC9914994; doi:10.3390/ijerph20032670)
Supplement: Supplementary file 1 [file ijerph-20-02670-s001.zip › ijerph-2165027-supplementary.pdf]

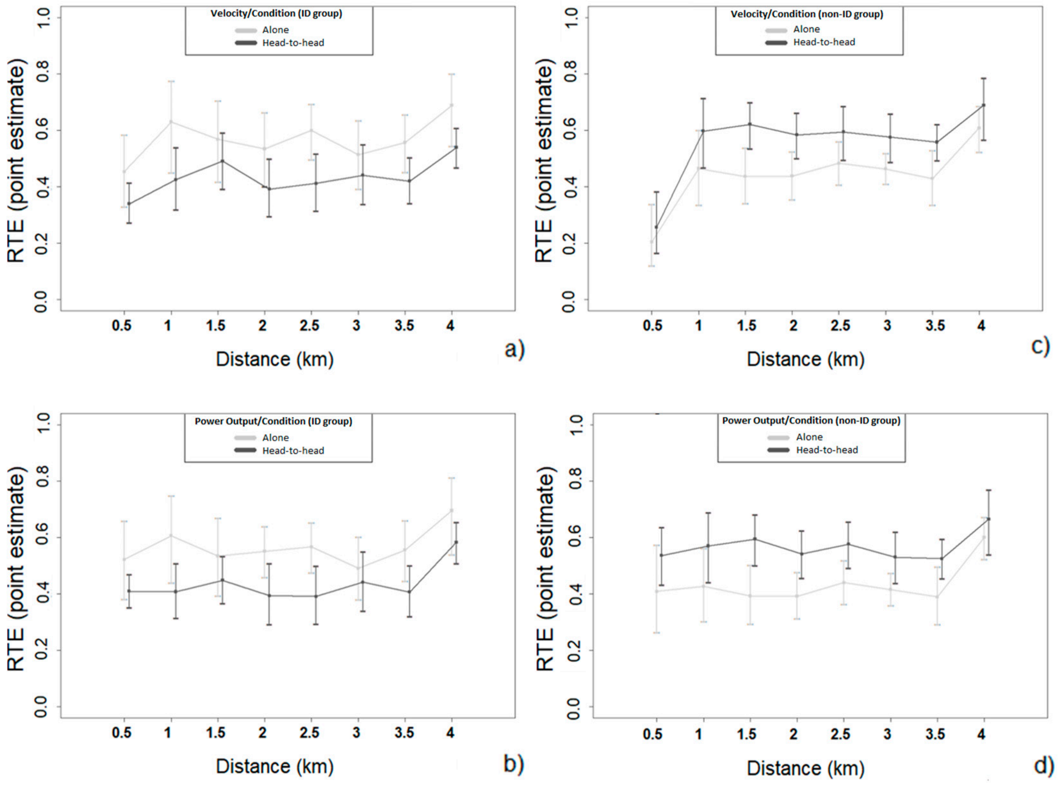

Figure S1. Relative effects and 95% confidence intervals for the velocity and power output of the ID (**a**,**b**) and non-ID group (**c**,**d**) during the 'alone' and 'head-to-head' condition
